# (Quinoline-2-carboxyl­ato-κ*O*)(quinoline-2-carb­oxy­lic acid-κ*O*)bis­(quinoline-2-carb­oxy­lic acid-κ^2^ *N*,*O*)potassium

**DOI:** 10.1107/S1600536810027510

**Published:** 2010-07-17

**Authors:** Seik Weng Ng

**Affiliations:** a Department of Chemistry, University of Malaya, 50603 Kuala Lumpur, Malaysia

## Abstract

The K atom in the title complex, [K(C_10_H_6_NO_2_)(C_10_H_7_NO_2_)_3_], lies on a twofold rotation axis that relates one *N*,*O*-chelating quinoline-2-carb­oxy­lic acid to the other; their N and O atoms are *cis* to each other in the distorted octa­hedral coordination geometry. The K atom is also coordinated by another monodentate quinoline-2-carb­oxy­lic acid; the acid is disordered with respect to a monodentate quinoline-2-carboxyl­ate anion; the acid and anion are linked by an O—H⋯O hydrogen bond. An O—H⋯N hydrogen bond links adjacent mol­ecules into a linear chain structure along the *a* axis.

## Related literature

For the crystal structure of quinoline-2-carb­oxy­lic acid, see: Dobrzyńska & Jerzykiewicz (2004 ▶).
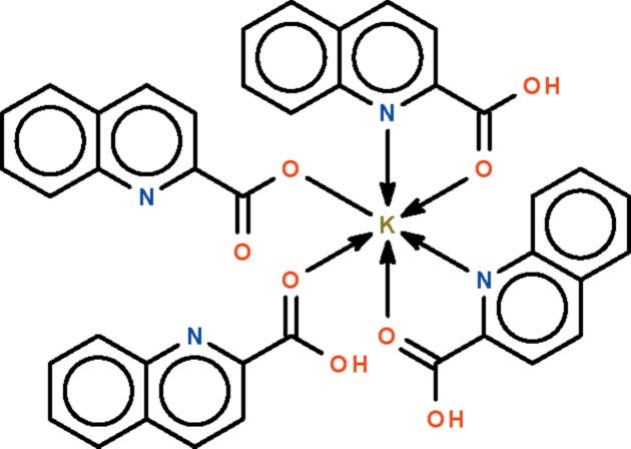


## Experimental

### 

#### Crystal data

[K(C_10_H_6_NO_2_)(C_10_H_7_NO_2_)_3_]
*M* *_r_* = 730.76
Orthorhombic, 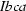

*a* = 17.8679 (10) Å
*b* = 18.3617 (10) Å
*c* = 20.5162 (11) Å
*V* = 6731.1 (6) Å^3^
*Z* = 8
Mo *K*α radiation
μ = 0.22 mm^−1^
*T* = 100 K
0.24 × 0.08 × 0.04 mm

#### Data collection

Bruker SMART APEX diffractometer
Absorption correction: multi-scan (*SADABS*; Sheldrick, 1996 ▶) *T*_min_ = 0.949, *T*_max_ = 0.991
40797 measured reflections
3888 independent reflections
3025 reflections with *I* > 2σ(*I*)
*R*_int_ = 0.075

#### Refinement

*R*[*F* ^2^ > 2σ(*F* ^2^)] = 0.035
*wR*(*F* ^2^) = 0.102
*S* = 1.01
3888 reflections
248 parameters
2 restraints
H atoms treated by a mixture of independent and constrained refinement
Δρ_max_ = 0.32 e Å^−3^
Δρ_min_ = −0.54 e Å^−3^

### 

Data collection: *APEX2* (Bruker, 2009 ▶); cell refinement: *SAINT* (Bruker, 2009 ▶); data reduction: *SAINT*; program(s) used to solve structure: *SHELXS97* (Sheldrick, 2008 ▶); program(s) used to refine structure: *SHELXL97* (Sheldrick, 2008 ▶); molecular graphics: *X-SEED* (Barbour, 2001 ▶); software used to prepare material for publication: *publCIF* (Westrip, 2010 ▶).

## Supplementary Material

Crystal structure: contains datablocks global, I. DOI: 10.1107/S1600536810027510/jh2182sup1.cif

Structure factors: contains datablocks I. DOI: 10.1107/S1600536810027510/jh2182Isup2.hkl

Additional supplementary materials:  crystallographic information; 3D view; checkCIF report

## Figures and Tables

**Table 1:** Hydrogen-bond geometry (Å, °)

*D*—H⋯*A*	*D*—H	H⋯*A*	*D*⋯*A*	*D*—H⋯*A*
O2—H2⋯N2^i^	0.85 (1)	1.84 (1)	2.671 (2)	167 (2)
O3—H3⋯O3^ii^	0.84 (1)	1.62 (1)	2.452 (2)	175 (6)

Symmetry codes: (i) 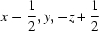; (ii) 
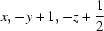
.

**Fig. 1.**
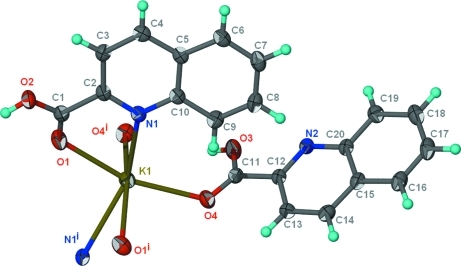
Thermal ellipsoid plot (Barbour, 2001) of a portion of the polymeric chain structure of K(C10H6NO2)(C10H7NO2)3 at the 70% probability level. Hydrogen atoms are drawn as spheres of arbitrary radius. Symmetry code: i = x, 1 – y, 1/2 – z.

## supplementary crystallographic information

### Comment

Quinoline-2-carboxylic acid exists as a 1:1 co-crystal of neutral quinoline-2-carboxylic acid and zwitterionic quinolinium-2-carboxylate, the two components being held together by O–H···O [2.566 (2) Å] and N–H···O [2.685 (2), 2.739 (2) Å] hydrogen bonds (Dobrzyńska & Jerzykiewicz, 2004). The potassium derivative formally exists as a co-crystal with three molecules of quinoline-2-carboxylic acid (Scheme I); however, the crystal structure is better interpreted in terms of the potassium atom being bis-*N*,*O*-chelated by two neutral acid molecules, and being coordinated by a third acid along with a carboxylate anion (Fig. 1); O–H···O and O–H···N hydrogen bonds link adjacent molecules into a linear chain structure.

The third acid and the carboxylate anion are disordered with respect to each other.

### Experimental

Quinoline-2-carboxylic acid (1 mmol, 0.17 g) and methyl-8-hydroxy quinoline (1 mmol, 0.16 g) were dissolved completely in warm acetonitrile; the solution was filtered into a clean beaker for the growth of colorless crystals.

As no potasium salt was used in the attempted co-crystallization of the organic compounds, the potassium in the crystal structure is better attributed to the presence of potassium quinoline-2-carboxylate present in the commercially procured carboxylic acid reagent.

### Refinement

Hydrogen atoms were placed in calculated positions (C–H 0.95 Å) and were included in the refinement in the riding model approximation, with *U*(H) set to 1.2*U*_eq_(C).

Of the two carboxylic acid hydrogen atoms, that connected to O2 lies on a general position and has full site-occupancy. That connected to O3 is near the Wyckoff 8*c* site so that the atom should have only half site-occupancy. The refinement of the two hydrogen atoms with a distance restraint of O–H 0.84±0.01 Å gave satisfactory temperature factors.

### Crystal data

**Table:**

[K(C_10_H_6_NO_2_)(C_10_H_7_NO_2_)_3_]	*F*(000) = 3024
*M**_r_* = 730.76	*D*_x_ = 1.442 Mg m^−^^3^
Orthorhombic, *I**b**c**a*	Mo *K*α radiation, λ = 0.71073 Å
Hall symbol: -I 2b 2c	Cell parameters from 8761 reflections
*a* = 17.8679 (10) Å	θ = 2.2–27.4°
*b* = 18.3617 (10) Å	µ = 0.22 mm^−^^1^
*c* = 20.5162 (11) Å	*T* = 100 K
*V* = 6731.1 (6) Å^3^	Prism, colorless
*Z* = 8	0.24 × 0.08 × 0.04 mm

### Data collection

**Table:**

Bruker SMART APEX diffractometer	3888 independent reflections
Radiation source: fine-focus sealed tube	3025 reflections with *I* > 2σ(*I*)
graphite	*R*_int_ = 0.075
ω scans	θ_max_ = 27.5°, θ_min_ = 2.0°
Absorption correction: multi-scan (*SADABS*; Sheldrick, 1996)	*h* = −23→23
*T*_min_ = 0.949, *T*_max_ = 0.991	*k* = −23→23
40797 measured reflections	*l* = −26→26

### Refinement

**Table:**

Refinement on *F*^2^	Primary atom site location: structure-invariant direct methods
Least-squares matrix: full	Secondary atom site location: difference Fourier map
*R*[*F*^2^ > 2σ(*F*^2^)] = 0.035	Hydrogen site location: inferred from neighbouring sites
*wR*(*F*^2^) = 0.102	H atoms treated by a mixture of independent and constrained refinement
*S* = 1.01	*w* = 1/[σ^2^(*F*_o_^2^) + (0.0526*P*)^2^ + 5.6795*P*] where *P* = (*F*_o_^2^ + 2*F*_c_^2^)/3
3888 reflections	(Δ/σ)_max_ = 0.001
248 parameters	Δρ_max_ = 0.32 e Å^−^^3^
2 restraints	Δρ_min_ = −0.54 e Å^−^^3^

### Fractional atomic coordinates and isotropic or equivalent isotropic displacement parameters (Å^2^)

**Table:**

	*x*	*y*	*z*	*U*_iso_*/*U*_eq_	Occ. (<1)
K1	0.21891 (3)	0.5000	0.2500	0.02083 (13)	
O1	0.11657 (7)	0.56758 (6)	0.17738 (6)	0.0256 (3)	
O2	0.03124 (6)	0.65608 (6)	0.16390 (6)	0.0232 (3)	
O3	0.43403 (7)	0.52894 (7)	0.30384 (5)	0.0257 (3)	
O4	0.32794 (6)	0.47403 (6)	0.33533 (5)	0.0245 (3)	
N1	0.17250 (7)	0.64593 (7)	0.28227 (6)	0.0183 (3)	
N2	0.45505 (7)	0.58087 (7)	0.42560 (6)	0.0171 (3)	
C1	0.09152 (8)	0.62730 (9)	0.19030 (7)	0.0190 (3)	
C2	0.12794 (8)	0.67785 (9)	0.23938 (7)	0.0175 (3)	
C3	0.11455 (9)	0.75361 (9)	0.23657 (7)	0.0193 (3)	
H3A	0.0804	0.7733	0.2057	0.023*	
C4	0.15184 (8)	0.79806 (9)	0.27927 (7)	0.0195 (3)	
H4	0.1460	0.8494	0.2769	0.023*	
C5	0.19914 (8)	0.76670 (8)	0.32701 (7)	0.0179 (3)	
C6	0.23871 (9)	0.80868 (9)	0.37409 (8)	0.0210 (3)	
H6	0.2348	0.8603	0.3738	0.025*	
C7	0.28244 (9)	0.77496 (9)	0.41982 (8)	0.0236 (4)	
H7	0.3086	0.8034	0.4511	0.028*	
C8	0.28908 (9)	0.69785 (9)	0.42095 (8)	0.0236 (4)	
H8	0.3192	0.6751	0.4532	0.028*	
C9	0.25237 (9)	0.65638 (9)	0.37590 (7)	0.0216 (3)	
H9	0.2572	0.6049	0.3769	0.026*	
C10	0.20707 (8)	0.68960 (8)	0.32758 (7)	0.0173 (3)	
C11	0.38543 (9)	0.50923 (8)	0.34589 (7)	0.0184 (3)	
C12	0.40191 (8)	0.53220 (8)	0.41573 (7)	0.0164 (3)	
C13	0.36025 (9)	0.49993 (9)	0.46699 (8)	0.0203 (3)	
H13	0.3214	0.4662	0.4577	0.024*	
C14	0.37688 (9)	0.51806 (9)	0.53016 (8)	0.0218 (3)	
H14	0.3505	0.4960	0.5652	0.026*	
C15	0.43344 (9)	0.56978 (9)	0.54274 (7)	0.0192 (3)	
C16	0.45424 (10)	0.59128 (9)	0.60692 (8)	0.0238 (4)	
H16	0.4301	0.5700	0.6435	0.029*	
C17	0.50871 (10)	0.64237 (10)	0.61602 (8)	0.0272 (4)	
H17	0.5220	0.6566	0.6590	0.033*	
C18	0.54557 (10)	0.67443 (10)	0.56217 (8)	0.0264 (4)	
H18	0.5833	0.7100	0.5693	0.032*	
C19	0.52729 (9)	0.65450 (9)	0.49966 (8)	0.0225 (3)	
H19	0.5523	0.6763	0.4638	0.027*	
C20	0.47121 (8)	0.60139 (8)	0.48864 (7)	0.0175 (3)	
H2	0.0082 (13)	0.6266 (11)	0.1393 (10)	0.057 (8)*	
H3	0.433 (2)	0.512 (3)	0.2662 (12)	0.051 (14)*	0.50

### Atomic displacement parameters (Å^2^)

**Table:**

	*U*^11^	*U*^22^	*U*^33^	*U*^12^	*U*^13^	*U*^23^
K1	0.0202 (2)	0.0198 (2)	0.0225 (2)	0.000	0.000	−0.00261 (19)
O1	0.0249 (6)	0.0250 (6)	0.0270 (6)	0.0012 (5)	−0.0050 (5)	−0.0085 (5)
O2	0.0218 (6)	0.0250 (6)	0.0227 (6)	−0.0007 (5)	−0.0062 (5)	−0.0032 (5)
O3	0.0272 (6)	0.0359 (7)	0.0140 (6)	−0.0066 (5)	0.0024 (5)	−0.0049 (5)
O4	0.0262 (6)	0.0257 (6)	0.0217 (6)	−0.0064 (5)	−0.0023 (5)	−0.0033 (5)
N1	0.0183 (6)	0.0201 (7)	0.0163 (6)	−0.0014 (5)	0.0018 (5)	−0.0021 (5)
N2	0.0176 (6)	0.0182 (7)	0.0154 (6)	0.0019 (5)	0.0001 (5)	−0.0012 (5)
C1	0.0183 (7)	0.0215 (8)	0.0171 (7)	−0.0022 (6)	0.0020 (6)	−0.0008 (6)
C2	0.0150 (7)	0.0214 (8)	0.0161 (7)	−0.0016 (6)	0.0026 (6)	−0.0026 (6)
C3	0.0175 (7)	0.0225 (8)	0.0180 (7)	0.0013 (6)	0.0008 (6)	0.0002 (6)
C4	0.0180 (7)	0.0195 (8)	0.0210 (7)	0.0012 (6)	0.0044 (6)	−0.0027 (6)
C5	0.0148 (7)	0.0207 (8)	0.0182 (7)	0.0000 (6)	0.0038 (6)	−0.0034 (6)
C6	0.0205 (8)	0.0215 (8)	0.0211 (7)	−0.0017 (6)	0.0036 (6)	−0.0051 (6)
C7	0.0212 (8)	0.0304 (9)	0.0191 (7)	−0.0024 (7)	0.0008 (6)	−0.0070 (7)
C8	0.0224 (8)	0.0305 (9)	0.0178 (7)	0.0036 (7)	−0.0023 (6)	−0.0014 (6)
C9	0.0224 (8)	0.0225 (8)	0.0198 (7)	0.0031 (6)	0.0012 (6)	−0.0018 (6)
C10	0.0168 (7)	0.0198 (8)	0.0154 (7)	−0.0005 (6)	0.0037 (5)	−0.0029 (6)
C11	0.0214 (8)	0.0169 (8)	0.0169 (7)	0.0021 (6)	−0.0018 (6)	−0.0009 (6)
C12	0.0160 (7)	0.0171 (7)	0.0162 (7)	0.0031 (6)	−0.0001 (5)	−0.0011 (6)
C13	0.0213 (8)	0.0196 (8)	0.0201 (7)	−0.0021 (6)	−0.0004 (6)	0.0008 (6)
C14	0.0245 (8)	0.0241 (9)	0.0168 (7)	0.0008 (7)	0.0020 (6)	0.0022 (6)
C15	0.0214 (8)	0.0198 (8)	0.0164 (7)	0.0056 (6)	−0.0011 (6)	−0.0020 (6)
C16	0.0285 (9)	0.0275 (9)	0.0155 (7)	0.0066 (7)	−0.0007 (6)	−0.0024 (6)
C17	0.0301 (9)	0.0299 (9)	0.0216 (8)	0.0075 (7)	−0.0070 (7)	−0.0094 (7)
C18	0.0231 (8)	0.0255 (9)	0.0306 (9)	0.0022 (7)	−0.0066 (7)	−0.0077 (7)
C19	0.0210 (8)	0.0226 (8)	0.0239 (8)	0.0006 (7)	−0.0009 (6)	−0.0027 (6)
C20	0.0176 (7)	0.0180 (8)	0.0170 (7)	0.0050 (6)	−0.0010 (6)	−0.0018 (6)

### Geometric parameters (Å, °)

**Table:**

K1—O4	2.6622 (12)	C6—C7	1.369 (2)
K1—O4^i^	2.6622 (12)	C6—H6	0.9500
K1—O1^i^	2.6653 (12)	C7—C8	1.421 (2)
K1—O1	2.6653 (12)	C7—H7	0.9500
K1—N1^i^	2.8820 (13)	C8—C9	1.365 (2)
K1—N1	2.8820 (13)	C8—H8	0.9500
O1—C1	1.2137 (19)	C9—C10	1.418 (2)
O2—C1	1.3163 (19)	C9—H9	0.9500
O2—H2	0.846 (10)	C11—C12	1.522 (2)
O3—C11	1.2765 (19)	C12—C13	1.418 (2)
O3—H3	0.836 (10)	C13—C14	1.371 (2)
O4—C11	1.2327 (19)	C13—H13	0.9500
N1—C2	1.323 (2)	C14—C15	1.411 (2)
N1—C10	1.3742 (19)	C14—H14	0.9500
N2—C12	1.320 (2)	C15—C20	1.423 (2)
N2—C20	1.3777 (19)	C15—C16	1.424 (2)
C1—C2	1.516 (2)	C16—C17	1.365 (2)
C2—C3	1.413 (2)	C16—H16	0.9500
C3—C4	1.370 (2)	C17—C18	1.414 (3)
C3—H3A	0.9500	C17—H17	0.9500
C4—C5	1.416 (2)	C18—C19	1.373 (2)
C4—H4	0.9500	C18—H18	0.9500
C5—C6	1.424 (2)	C19—C20	1.416 (2)
C5—C10	1.423 (2)	C19—H19	0.9500
			
O4—K1—O4^i^	85.93 (5)	C6—C7—H7	119.7
O4—K1—O1^i^	92.93 (4)	C8—C7—H7	119.7
O4^i^—K1—O1^i^	162.38 (3)	C9—C8—C7	120.31 (15)
O4—K1—O1	162.38 (4)	C9—C8—H8	119.8
O4^i^—K1—O1	92.93 (4)	C7—C8—H8	119.8
O1^i^—K1—O1	93.36 (5)	C8—C9—C10	120.50 (15)
O4—K1—N1^i^	101.25 (4)	C8—C9—H9	119.7
O4^i^—K1—N1^i^	103.07 (4)	C10—C9—H9	119.7
O1^i^—K1—N1^i^	59.88 (4)	N1—C10—C9	118.60 (14)
O1—K1—N1^i^	96.14 (4)	N1—C10—C5	122.02 (14)
O4—K1—N1	103.07 (4)	C9—C10—C5	119.38 (14)
O4^i^—K1—N1	101.25 (4)	O4—C11—O3	126.64 (14)
O1^i^—K1—N1	96.14 (4)	O4—C11—C12	118.16 (14)
O1—K1—N1	59.88 (4)	O3—C11—C12	115.20 (13)
N1^i^—K1—N1	146.55 (5)	N2—C12—C13	123.15 (14)
C1—O1—K1	123.47 (10)	N2—C12—C11	118.12 (13)
C1—O2—H2	112.7 (17)	C13—C12—C11	118.73 (13)
C11—O3—H3	120 (3)	C14—C13—C12	119.07 (15)
C11—O4—K1	129.15 (10)	C14—C13—H13	120.5
C2—N1—C10	117.50 (13)	C12—C13—H13	120.5
C2—N1—K1	115.60 (10)	C13—C14—C15	119.46 (15)
C10—N1—K1	124.64 (10)	C13—C14—H14	120.3
C12—N2—C20	118.69 (13)	C15—C14—H14	120.3
O1—C1—O2	125.07 (14)	C14—C15—C20	118.15 (14)
O1—C1—C2	122.68 (14)	C14—C15—C16	122.87 (15)
O2—C1—C2	112.25 (13)	C20—C15—C16	118.97 (15)
N1—C2—C3	124.42 (14)	C17—C16—C15	120.21 (16)
N1—C2—C1	115.38 (14)	C17—C16—H16	119.9
C3—C2—C1	120.19 (13)	C15—C16—H16	119.9
C4—C3—C2	118.55 (14)	C16—C17—C18	120.75 (15)
C4—C3—H3A	120.7	C16—C17—H17	119.6
C2—C3—H3A	120.7	C18—C17—H17	119.6
C3—C4—C5	119.35 (15)	C19—C18—C17	120.52 (16)
C3—C4—H4	120.3	C19—C18—H18	119.7
C5—C4—H4	120.3	C17—C18—H18	119.7
C4—C5—C6	123.06 (15)	C18—C19—C20	120.06 (16)
C4—C5—C10	118.02 (14)	C18—C19—H19	120.0
C6—C5—C10	118.92 (14)	C20—C19—H19	120.0
C7—C6—C5	120.24 (15)	N2—C20—C19	119.10 (14)
C7—C6—H6	119.9	N2—C20—C15	121.42 (14)
C5—C6—H6	119.9	C19—C20—C15	119.47 (14)
C6—C7—C8	120.63 (15)		
			
O4—K1—O1—C1	−19.8 (2)	C6—C7—C8—C9	0.6 (2)
O4^i^—K1—O1—C1	−105.57 (12)	C7—C8—C9—C10	−0.2 (2)
O1^i^—K1—O1—C1	90.90 (12)	C2—N1—C10—C9	177.15 (13)
N1^i^—K1—O1—C1	150.95 (12)	K1—N1—C10—C9	−20.70 (18)
N1—K1—O1—C1	−4.26 (11)	C2—N1—C10—C5	−3.3 (2)
O4^i^—K1—O4—C11	45.39 (12)	K1—N1—C10—C5	158.83 (10)
O1^i^—K1—O4—C11	−152.23 (13)	C8—C9—C10—N1	178.53 (14)
O1—K1—O4—C11	−41.5 (2)	C8—C9—C10—C5	−1.0 (2)
N1^i^—K1—O4—C11	147.92 (13)	C4—C5—C10—N1	2.2 (2)
N1—K1—O4—C11	−55.23 (13)	C6—C5—C10—N1	−177.82 (13)
O4—K1—N1—C2	167.93 (10)	C4—C5—C10—C9	−178.25 (14)
O4^i^—K1—N1—C2	79.54 (11)	C6—C5—C10—C9	1.7 (2)
O1^i^—K1—N1—C2	−97.62 (10)	K1—O4—C11—O3	−53.0 (2)
O1—K1—N1—C2	−7.30 (10)	K1—O4—C11—C12	127.14 (12)
N1^i^—K1—N1—C2	−56.46 (10)	C20—N2—C12—C13	0.2 (2)
O4—K1—N1—C10	5.47 (12)	C20—N2—C12—C11	−179.12 (13)
O4^i^—K1—N1—C10	−82.91 (11)	O4—C11—C12—N2	−167.51 (14)
O1^i^—K1—N1—C10	99.93 (11)	O3—C11—C12—N2	12.7 (2)
O1—K1—N1—C10	−169.75 (13)	O4—C11—C12—C13	13.2 (2)
N1^i^—K1—N1—C10	141.09 (12)	O3—C11—C12—C13	−166.67 (14)
K1—O1—C1—O2	−165.66 (11)	N2—C12—C13—C14	−2.0 (2)
K1—O1—C1—C2	14.9 (2)	C11—C12—C13—C14	177.30 (14)
C10—N1—C2—C3	1.0 (2)	C12—C13—C14—C15	1.8 (2)
K1—N1—C2—C3	−162.79 (11)	C13—C14—C15—C20	0.1 (2)
C10—N1—C2—C1	179.71 (12)	C13—C14—C15—C16	−179.71 (15)
K1—N1—C2—C1	15.95 (16)	C14—C15—C16—C17	−179.08 (15)
O1—C1—C2—N1	−21.4 (2)	C20—C15—C16—C17	1.1 (2)
O2—C1—C2—N1	159.12 (13)	C15—C16—C17—C18	−0.3 (2)
O1—C1—C2—C3	157.39 (15)	C16—C17—C18—C19	−0.2 (3)
O2—C1—C2—C3	−22.09 (19)	C17—C18—C19—C20	−0.1 (2)
N1—C2—C3—C4	2.5 (2)	C12—N2—C20—C19	−178.94 (14)
C1—C2—C3—C4	−176.21 (14)	C12—N2—C20—C15	1.8 (2)
C2—C3—C4—C5	−3.5 (2)	C18—C19—C20—N2	−178.40 (14)
C3—C4—C5—C6	−178.65 (14)	C18—C19—C20—C15	0.9 (2)
C3—C4—C5—C10	1.3 (2)	C14—C15—C20—N2	−1.9 (2)
C4—C5—C6—C7	178.70 (15)	C16—C15—C20—N2	177.87 (14)
C10—C5—C6—C7	−1.3 (2)	C14—C15—C20—C19	178.81 (14)
C5—C6—C7—C8	0.1 (2)	C16—C15—C20—C19	−1.4 (2)

Symmetry codes: (i) *x*, −*y*+1, −*z*+1/2.

### Hydrogen-bond geometry (Å, °)

**Table:**

*D*—H···*A*	*D*—H	H···*A*	*D*···*A*	*D*—H···*A*
O2—H2···N2^ii^	0.85 (1)	1.84 (1)	2.671 (2)	167 (2)
O3—H3···O3^i^	0.84 (1)	1.62 (1)	2.452 (2)	175 (6)

Symmetry codes: (ii) *x*−1/2, *y*, −*z*+1/2; (i) *x*, −*y*+1, −*z*+1/2.
